# Decoding Dishevelled-Mediated Wnt Signaling in Vertebrate Early Development

**DOI:** 10.3389/fcell.2020.588370

**Published:** 2020-09-30

**Authors:** De-Li Shi

**Affiliations:** Developmental Biology Laboratory, CNRS-UMR 7622, IBPS, Sorbonne University, Paris, France

**Keywords:** Wnt signaling, Dishevelled, Wnt/ß-catenin, Wnt/PCP, convergence and extension, mouse, *Xenopus*, zebrafish

## Abstract

Dishevelled proteins are key players of Wnt signaling pathways. They transduce Wnt signals and perform cellular functions through distinct conserved domains. Due to the presence of multiple paralogs, the abundant accumulation of maternal transcripts, and the activation of distinct Wnt pathways, their regulatory roles during vertebrate early development and the mechanism by which they dictate the pathway specificity have been enigmatic and attracted much attention in the past decades. Extensive studies in different animal models have provided significant insights into the structure-function relationship of conserved Dishevelled domains in Wnt signaling and the implications of Dishevelled isoforms in early developmental processes. Notably, intra- and inter-molecular interactions and Dishevelled dosage may be important in modulating the specificity of Wnt signaling. There are also distinct and redundant functions among Dishevelled isoforms in development and disease, which may result from differential spatiotemporal expression patterns and biochemical properties and post-translational modifications. This review presents the advances and perspectives in understanding Dishevelled-mediated Wnt signaling during gastrulation and neurulation in vertebrate early embryos.

## Introduction

Wnt signaling plays critical roles in a wide variety of biological processes, including embryonic axis formation, cell proliferation, differentiation and migration, polarity establishment, and stem cell self-renewal (Steinhart and Angers, 2018; Wiese et al., 2018). Upon stimulation by Wnt ligands, membrane receptors (Frizzled) and co-receptors (LRP5/6, glypican-3/4, ROR, and RYK) assemble into complexes to activate divergent pathways (Niehrs, 2012; Green et al., 2014; Stricker et al., 2017). The activity of Frizzled receptors is further controlled by an auxiliary regulatory system involving RSPO1-4, LGR4/5/6 and ZNRF3/RNF43 (Jiang and Cong, 2016; Lehoczky and Tabin, 2018). The canonical Wnt pathway (Wnt/ß-catenin) regulates target gene transcription through stabilization and nuclear accumulation of ß-catenin by inhibition of its destruction complex, consisting of Axin-GSK3ß-APC, whereas the non-canonical Wnt pathway (Wnt/planar cell polarity or Wnt/PCP) is implicated in polarized cellular orientation and asymmetric cell movements through activation of major regulators of the cytoskeleton. Aberrant signaling of both pathways leads to tumorigenesis and metastasis of multiple cancer types, as well as human birth defects (Clevers and Nusse, 2012; Butler and Wallingford, 2017; Humphries and Mlodzik, 2018), but how they are regulated in development and disease remains elusive.

Dishevelled (Dvl or Dsh in *Drosophila*) is a family of proteins that function as common intracellular conductors of both Wnt/ß-catenin and Wnt/PCP pathways (Boutros and Mlodzik, 1999; Wallingford and Habas, 2005; Gao and Chen, 2010). *Drosophila dsh* alleles were first identified in genetic mutants with disruptions of hair and bristle polarity (Wallingford and Habas, 2005). Vertebrates possess three highly conserved *Dvl* genes. Extensive studies in mouse, *Xenopus* and zebrafish have revealed their critical roles in germ layer specification and morphogenetic movements, which require Wnt/ß-catenin and Wnt/PCP signaling, respectively. There is accumulating evidence that Dvl isoforms display both distinct and redundant functions (Gentzel and Schambony, 2017). However, a number of important questions regarding Dvl-mediated Wnt signaling during development remain enigmatic (Mlodzik, 2016), such as Dvl-regulated switch of distinct Wnt pathways, the specific functions of Dvl isoforms in Wnt signaling and development, the post-translational modifications of Dvl functions, and the maternal contributions of Dvl to early developmental events. Fortunately, structure-function and mutational analyses have significantly advanced our understanding of Dvl-regulated Wnt signaling in development and disease. This review focuses on progresses made in this fascinating research field by using complementary vertebrate animal models.

## Dvl Functional Domains in Wnt Signaling

**FIGURE 1:**
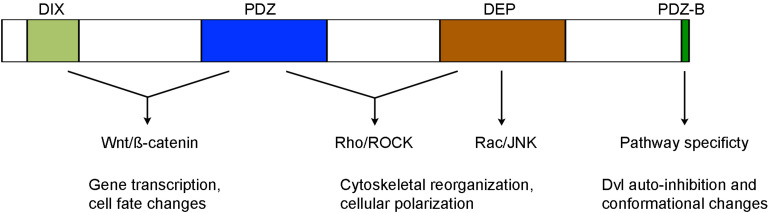
Structure-function relationship of conserved Dvl domains in the activation of Wnt/ß-catenin and Wnt/PCP signaling (see text for details).

Dvl proteins contain several highly conserved domains required for activating different Wnt pathways, including in particular the N-terminal DIX (Dishevelled and aXin) domain, the central PDZ (Post-synaptic density protein-95, Disk large tumor suppressor, Zonula occludens-1) domain, and the C-terminal DEP (Dishevelled, Egl-10 and Pleckstrin) domain (Figure 1). The DIX domain is involved in Wnt-induced dynamic Dvl homo- and hetero-oligomerization that is important for Wnt/ß-catenin signaling (Kishida et al., 1999; Kan et al., 2020; Ma et al., 2020). The PDZ domain interacts with a conserved KTxxxW motif located immediately after the seventh transmembrane domain of Frizzled receptors (Umbhauer et al., 2000; Wong et al., 2003), and with a wide variety of binding partners that function either as agonists or antagonists of Wnt signaling (Wallingford and Habas, 2005; Sharma et al., 2018). Biochemical and functional analyses suggest that it participates in both Wnt/ß-catenin and Wnt/PCP signaling (Habas et al., 2001; Lee et al., 2015). The DEP domain plays a major role in Dvl membrane recruitment by Frizzled receptors (Axelrod et al., 1998; Rothbächer et al., 2000; Wong et al., 2000; Pan et al., 2004; Park et al., 2005). It functions in the Wnt/PCP pathway either with the PDZ domain to activate Rho/ROCK or by direct interaction with Rac to trigger JNK activation. However, more recent evidence suggests that Dvl dimerization triggered by the N-terminal region of DEP domain is required for Wnt/ß-catenin signaling (Gammons et al., 2016a,b; Paclíková et al., 2017).

The C-terminal region beyond the DEP domain also shows evolutionary conservation but displays unique features among Dvl isofroms. Specifically, Dvl3 contains histidine-single amino acid repeats required for Wnt5a-stimulated activation of nuclear factor of activated T cells (NF-AT) and possesses proline-rich domains likely involved in the interaction with other Dvl isoforms (Ma et al., 2010; Wang and Malbon, 2012). The extreme 13 amino acids that are conserved in all Dvl isoforms bind to the third intracellular loop of Frizzled receptors and stabilize Frizzled-Dvl interaction in Wnt/ß-catenin signaling (Tauriello et al., 2012). Moreover, the last 3 residues represent a type II PDZ-binding (PDZ-B) motif that can occupy the peptide-binding pocket of the PDZ domain, inducing Dvl to adopt a closed conformation and an auto-inhibited state (Lee et al., 2015; Qi et al., 2017). Dvl variants with an opened conformation show efficient membrane recruitment and reduced activity in Wnt/ß-catenin signaling but display increased activity in Wnt/PCP signaling (Qi et al., 2017; Harnoš et al., 2019). The function of Dvl C-terminal region in Wnt signaling is further demonstrated in autosomal-dominant Robinow syndrome caused by *de novo* frameshift mutations in human *DVL1* and *DVL3* genes, which delete and replace the C-terminal region after the DEP domain (Bunn et al., 2015; White et al., 2015, 2016; Danyel et al., 2018). *In vitro* analysis suggests that DVL1 lacking the C-terminal region displays reduced activity in Wnt/ß-catenin signaling (Bunn et al., 2015). These findings suggest an importance of the C-terminus in intra- or inter-molecular interaction, which may be subjected to regulation by other partners to switch pathway specificity. Indeed, recent studies show that casein kinase 1ε (CK1ε) and NIMA-related kinase 2 (NEK2) function as scaffold proteins and regulate the dynamics of Dvl conformational changes by phosphorylation of the PDZ domain and modulation of its interaction with the extreme C-terminal tail (Harnoš et al., 2019; Hanáková et al., 2019).

## Dvl Dosage Effect on Morphogenetic Movements and Cell Fate Specification During Development in Mice

The three *Dvl* genes (*Dvl1*, *Dvl2*, and *Dvl3*) in mice are broadly expressed throughout early development. Extensive analyses of mutant phenotypes have uncovered both unique and redundant functions for these genes. Mice deficient in *Dvl1* show reduced social interaction and abnormal sensorimotor gating (Lijam et al., 1997). This abnormal behavior is caused by defective Wnt/ß-catenin signaling that may impair central nervous system functions (Belinson et al., 2016). Mice deficient in *Dvl2* exhibit more severe phenotypes, with defective cardiac morphogenesis, somite segmentation, and neural tube closure (Hamblet et al., 2002). *Dvl3* functions redundantly with *Dvl1* and *Dvl2* in several processes, including cardiac outflow tract, cochlea and neural tube development (Etheridge et al., 2008). These works reveal a sensitivity of Wnt/PCP signaling to *Dvl* dosage because most defective phenotypes in *Dvl* mutants are related to impaired Wnt/PCP signaling, in particular the defective outflow tract morphogenesis (Sinha et al., 2012). Thus, Dvl isoforms are critically required for morphogenetic movements. Particularly, they mediate Wnt/PCP in CE movements during neurulation. Dvl2 plays a predominant role in neural tube closure, but Dvl1 and Dvl3 are also involved in this process (Wang et al., 2006). The functional importance of Dvl isoforms in neural tube formation has been confirmed by the identification of rare mutations in all three human *DVL* genes, which disturb normal functions of DVL isoforms in non-canonical Wnt signaling and cause neural tube defects (De Marco et al., 2013; Liu et al., 2020). By comparison, low levels of Dvl expression from a single allele may be sufficient to normally support those developmental processes triggered by the Wnt/ß-catenin pathway (Soares et al., 2005; Wynshaw-Boris, 2012). However, deletion of all six *Dvl* alleles causes absence of mesoderm gene expression and mesoderm formation that are dependent on Wnt/ß-catenin signaling (Ngo et al., 2020).

## Distinct and Redundant Dvl Functions During *Xenopus* Development

Dvl function in vertebrates was first studied during *Xenopus* development. Overexpression of Dvl2 (Xdsh) in the ventral region of early embryos induced the formation of a complete secondary axis reminiscent of activation of maternal Wnt/ß-catenin signaling (Sokol et al., 1995). However, dorsal overexpression of Xdd1, a truncated form of Dvl2 that lacks the PDZ domain and interferes with Wnt/ß-catenin signaling triggered by Wnt ligands, did not affect dorsoventral axis formation (Sokol, 1996). Because maternal Wnt/ß-catenin signaling is required for dorsal fate specification by activating the transcription of target genes in the Spemann organizer (Carron and Shi, 2016), the absence of an inhibitory effect by Xdd1 implies that Dvl function may be dispensable for the activation of maternal Wnt/ß-catenin signaling. Consistently, simultaneous depletion of maternally expressed *Dvl2* and *Dvl3* from oocytes did not affect the expression of maternal Wnt/ß-catenin target genes and the formation of dorsal axis (Tadjuidje et al., 2011). However, it is possible that low levels of Dvl proteins are still present in the oocytes due to incomplete depletion of maternal *Dvl* mRNA. Thus, the requirement of maternal *Dvl* for dorsal axis formation in *Xenopus* requires a complete loss-of-function study. Nevertheless, a recent study suggests that activation of maternal Wnt/ß-catenin pathway and formation of dorsal axis may be achieved through a Dvl-independent mechanism (Yan et al., 2018). During organogenesis, it seems that Dvl isoforms display less functional redundancy in developmental processes that involve Wnt/ß-catenin signaling, which may be due to their differential expression patterns. For example, Dvl1 and Dvl2 are required for neural crest cell specification and somite segmentation, while Dvl3 maintains gene expression in the myotome (Gray et al., 2009).

Maternal Dvl2 and Dvl3 likely display distinct and redundant functions during CE movements. In these coordinated processes, lateral cells converge toward the dorsal region to narrow the germ layers, while dorsal midline cells undergo mediolateral intercalation by polarized protrusive behaviors to lengthen the embryo along the anteroposterior axis (Keller and Sutherland, 2020). Previous studies show that Dvl regulates CE movements through Wnt/PCP signaling (Djiane et al., 2000; Tada and Smith, 2000; Habas et al., 2001, 2003; Wallingford and Harland, 2001). Moreover, inhibition of maternal Dvl2 or Dvl3 function suggests that they exhibit a non-redundant but an additive effect on CE movements (Tadjuidje et al., 2011). More recent works reveal that Dvl1, but not Dvl2 or Dvl3, activates the Wnt/Ca^2+^ pathway (another branch of the non-canonical pathway) during CE movements (Gentzel et al., 2015), further supporting the distinct functions of Dvl isoforms in morphogenetic movements. As in mice, Dvl function is also required for neural tube closure by regulating CE movements of the midline and coordinating polarity among epithelial cells (Wallingford and Harland, 2002; Seo et al., 2017). In addition, Dvl2-mediated activation of Rac1 through the DEP domain also controls the protrusive activity of neural crest cells during migration (Kratzer et al., 2020). Because cellular polarization is tightly dependent on the asymmetric activation of Wnt/PCP signaling, both reduced and increased levels of Dvl disrupts asymmetric movements. However, the cellular behaviors are completely different. Increasing the activity of Dvl perturbs cell polarity by randomizing the formation of cellular protrusions, whereas reducing the activity of Dvl prevents cellular protrusions (Wallingford et al., 2000; Cheng et al., 2017).

## Maternal Contributions of Dvl Proteins to Axis Patterning and Morphogenetic Processes During Zebrafish Development

The zebrafish genome contains five *Dvl* paralogs: *dvl1a*, *dvl1b*, *dvl2*, *dvl3a*, and *dvl3b*. In the early embryos, *dvl2* and *dvl3a* are maternally expressed and represent about 98% of the total pool, whereas the transcript levels of the other *Dvl* genes are negligible (Harvey et al., 2013). Knockout of *Dvl* genes reveal both distinct and redundant functions in embryonic axis specification and morphogenetic movements (Xing et al., 2018). Maternal-zygotic mutants for *dvl1a*, *dvl1b*, *dvl3a*, and *dvl3b* are phenotypically normal and fertile. In sharp contrast, maternal-zygotic *dvl2* mutants display strongly impaired CE movements during gastrulation and develop severe craniofacial defects with a “bulldog” facial phenotype, reminiscent of impaired Wnt/PCP signaling in midline structures (Kimmel et al., 2001). Zygotic *dvl2* mutant embryos are essentially normal, but only about half of them can survive to adulthood, and all male individuals show absence of courtship behavior. This suggests that there may be defects in central nervous system functions as mice *Dvl1* mutants (Lijam et al., 1997). The highest level of *dvl2* expression during early development may explain at least partly the most severe phenotypes of *dvl2* mutants.

The specification of dorsal axis in zebrafish also requires maternal ß-catenin signaling to trigger the expression of organizer genes (Kelly et al., 2000; Bellipanni et al., 2006; Fuentes et al., 2020). However, the involvement of upstream regulators has not been conclusively established. Importantly, maternal-zygotic *dvl2* and *dvl3a* double mutants, which are unresponsive to stimulation by Wnt ligands, show normal specification of dorsal cell fate, suggesting that components of Wnt signaling upstream of ß-catenin may be dispensable for its stabilization. The activation of maternal Wnt/ß-catenin signaling independent of Dvl activity is further confirmed in zebrafish *huluwa* mutants. Maternal depletion of *huluwa* impairs Wnt/ß-catenin signaling and causes loss of dorso-anterior structures. Mechanistically, Huluwa protein accumulates in the cell membrane at the dorsal region and functions independently of Wnt ligands and Frizzled receptors to promote tankyrase-mediated degradation of Axin, thereby stabilizing ß-catenin (Yan et al., 2018).

**FIGURE 2:**
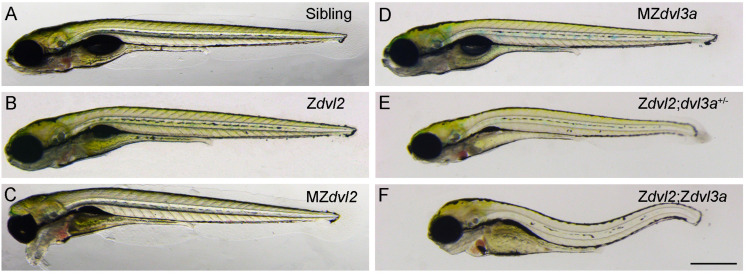
Maternal and zygotic Dvl dosage effect on embryonic axis elongation in zebrafish embryos at 5 days post-fertilization (Xing et al., 2018). **(A)** Wild-type sibling embryo. **(B)** Zygotic *dvl2* mutant. **(C)** The “bulldog” facial phenotype of maternal-zygotic *dvl2* mutant with cyclopia. **(D)** Maternal-zygotic *dvl3a* mutant. **(E)** Triallelic *dvl2* and *dvl3a* mutant. **(F)** Zygotic *dvl2* and *dvl3a* double mutant. Scale bar **(A–F)**: 400 μm.

Mutational analyses of Dvl functions also reveal a major contribution of maternal Dvl to zygotic events and confirm the importance of Dvl dosage in Wnt/PCP signaling (Figure 2). This dosage effect is particularly reflected by the requirement of Dvl2 and Dvl3a for CE movements. Although Dvl2 plays a predominant role, Dvl3a exerts a strong synergistic effect on the loss of Dvl2 function, and progressive removal of Dvl2 and Dvl3a maternal or zygotic products increasingly aggravates CE defects and reduces the elongation of anteroposterior axis. Furthermore, zygotic *dvl2* and *dvl3a* double mutants only display a shortened body length. However, maternal-zygotic *dvl2* and *dvl3a* double mutants show most strongly impaired CE movements and completely lack axis extension. They also develop severe trunk and posterior deficiencies associated with down-regulation of zygotic Wnt/ß-catenin target genes (Xing et al., 2018). Because zygotic Wnt/ß-catenin signaling has an opposite effect with respect to maternal Wnt/ß-catenin signaling and functions to specify the posterior region (Carron and Shi, 2016), these findings highlight the importance of maternal Dvl in setting up zygotic morphogenetic and patterning processes. They support the view that maternally expressed gene products perform essential functions after zygotic genome activation (Marlow, 2020; Solnica-Krezel, 2020). Thus, both maternal and zygotic Dvl dosages are important for proper cell movements and embryonic axis patterning that occur during gastrulation.

## Perspectives

Dvl conserved domains in Wnt pathways have attracted much attention. Although the function of DIX, PDZ, and DEP domains is relatively understood, how they cooperate to switch pathway specificity remains elusive. Detailed analysis of other conserved domains or isoform-specific regions, such as the basic region preceding the PDZ domain, the proline-rich region and histidine-single amino acid repeats in the C-terminal region beyond the DEP domain, and the extreme C-terminus, may provide insights into Dvl-mediated signal transduction. Because Dvl post-translational modifications, in particular phosphorylation and ubiquitination, and Dvl interaction partners are important for subcellular localizations and specific functions of Dvl proteins (Sharma et al., 2018; Harrison et al., 2020), it is of interest to understand how these modulate Dvl activity and dictate signaling outcomes in key developmental processes. Indeed, dysregulation of Dvl phosphorylation impairs both Wnt/ß-catenin and Wnt/PCP signaling during zebrafish and *Xenopus* embryogenesis (Shimizu et al., 2014; Rauschenberger et al., 2017). The specificity of Dvl isoforms also merits investigations because tissue-specific expression patterns and differential biochemical properties may contribute to their particular functions. Another intriguing question is the sensibility of Wnt/PCP signaling, but not Wnt/ß-catenin signaling, to Dvl dosage, which is observed in all vertebrates. It suggests that Wnt/PCP-dependent developmental processes critically require Dvl function to activate downstream effectors. Consequently, moderate diminution of Dvl dosage could significantly affect polarized cellular behaviors and cell polarity. By comparison, ß-catenin may be stabilized independently of upstream Wnt signaling components, and target genes of Wnt/ß-catenin signaling may be regulated by other factors, at least during dorsal fate specification in zebrafish and *Xenopus* (Li et al., 2015; Yan et al., 2018). Thus, tissue morphogenesis regulated by Wnt/PCP signaling is more sensitive to Dvl dysfunction, as a result, many human congenital disorders, such as neural tube defects and Robinow syndrome, are associated with mutations in *DVL* genes.

## Author Contributions

D-LS performed the literature analysis, prepared the figures, and wrote the manuscript.

## Conflict of Interest

The authors declare that the research was conducted in the absence of any commercial or financial relationships that could be construed as a potential conflict of interest.
